# 18-year-old Female with a Change in Mental Status

**DOI:** 10.5811/cpcem.2017.2.33875

**Published:** 2017-02-21

**Authors:** Laura J Bontempo, Andrew Crouter, Danya Khoujah, Zachary DW Dezman

**Affiliations:** * University of Maryland School of Medicine, Department of Emergency Medicine, Baltimore, MD; † University of Maryland Medical Center, Department of Emergency Medicine, Baltimore, MD

## CASE PRESENTATION

An 18-year-old female presented to the emergency department (ED) with confusion and “abnormal behavior.” Her family stated that she’d had increasingly abnormal speech for one week, word-finding difficulties, and then required frequent redirection to complete tasks. Two weeks prior to presentation, the patient was involved in a motor vehicle crash (MVC). The vehicles were moving slowly and sustained little damage. The airbags did not deploy and the patient was belted. She subsequently developed a series of headaches that persisted for a week and then resolved. Two days before presenting to our ED, she was seen at another ED for intermittent, crampy abdominal pain accompanied by vomiting, fevers to 38.9°C, and headache. She was diagnosed with a urinary tract infection (UTI) and discharged, but she did not take the nitrofurantoin she was prescribed. These symptoms continued on the day of presentation to our ED.

She had a past medical history of sickle cell trait. She did not take any medications and she had no drug allergies. Her family history was notable for epilepsy and systemic lupus erythematosus. She denied any tobacco, alcohol, or illicit drug use. She was a recent high school graduate.

She was alert and in no acute distress on physical exam. She was afebrile (37.1°C) with a heart rate of 90 beats/minute, blood pressure 135/85 mmHg, and her oxygen saturation was 99% while breathing room air. She weighed 100.5 kg and was five feet, six inches in height, had a body mass index of 36.9 kg/m^2^, and was well developed and well nourished. Her head was normocephalic and atraumatic, and mucus membranes were dry. Pupils were equal, round and reactive to light and accommodation; the extra ocular movements were normal. Sclera were anicteric and fundi were without papilledema. The neck was supple and without lymphadenopathy or carotid bruits. Her lungs had coarse breath sounds bilaterally but no wheezes, crackles, or rhonchi. There were no retractions or increased work of breathing. Heart was regular, rate and rhythm, without murmurs, rubs or gallops. The abdomen was soft with normal bowel sounds and without distention, tenderness, rebound or guarding. There was no costovertebral angle tenderness. The extremities had no edema, had 2+ pulses and were without tenderness or deformity.

Neurologic examination showed cranial nerves II–XII intact, 5/5 strength throughout all extremities, normal muscle bulk/tone and intact sensation. Her speech was clear. The patient was noted to be withdrawn and have a flat affect. She followed commands, answered most questions appropriately but occasionally was confused. She was oriented to self but not to place or time.

Initial laboratory results are shown in Tables 1–3. Because of her mental status changes and history of fever, a lumbar puncture was done. Her opening pressure was 12 cmH_2_0. The results are shown in Table 4. A diagnostic test was then performed, which confirmed the diagnosis.

## CASE DISCUSSION

When I first read this case, I realized that there was a lot of information to sort through. Specifically, in the history of present illness (HPI), there were several keywords that drew my attention:

18 year-old female presenting with confusion and “abnormal behavior” - abnormal speech, word-finding difficulties, and need for frequent redirection of tasks, for approximately one week.

She also had intermittent crampy abdominal pain, vomiting, intermittent fevers to 102°F, and headache for three days.

She was seen two days prior for similar complaints and diagnosed with a UTI, but did not fill her prescription.

Fourteen days prior she was involved in a minor MVC and subsequently developed headaches that persisted for a week and then resolved.

The physical exam was most remarkable for just a few findings: she had dry mucous membranes and a flat, withdrawn affect; she had ability to follow commands and answer most questions appropriately; but occasionally she was confused and oriented to self but not to place or time.

In an attempt to analyze this information, I went back to my mnemonic for mental status change, ”I WATCH DEATH.” Infectious Withdrawal Acute metabolic disorder Trauma Central nervous system (CNS) pathology Hypoxia Deficiencies Endocrinopathies Acute vascular Toxins, substance use, medication Heavy metals

**I**nfectious etiology: Here the differential includes CNS infections (meningitis, encephalitis) and any systemic infection. The patient had a history of fever but was afebrile in the ED, and her white blood cell count (WBC), C-reactive protein and sedimentation rate were all normal. Her urinalysis was inconsistent with an infectious source. The cerebrospinal fluid (CSF), however, did have white blood cells present, but only 12, which is atypical of an acute infection. So, for now, I will keep infection on my list of possibilities but continue to look elsewhere for answers.

**W**ithdrawal: Alcohol and benzodiazepine withdrawal can cause mental status changes. Given the patient’s lack of substance abuse history, coupled with a duration of symptoms greater than one week with gradual worsening, these causes are significantly less likely.

**A**cute metabolic disorders: Disturbances in sodium, calcium, or magnesium levels, as well as hepatic and renal failure, can present with altered mental status. The normal serum electrolyte levels, and normal renal and liver functions, excluded this possibility.

**T**rauma: I know that the patient had a minor MVC two weeks prior to presentation, so the differential must include post-concussive syndrome, as well as a possible subdural, epidural, or subarachnoid hemorrhage. Dr. Crouter described a minor MVC, which would have a low likelihood of causing an intracranial hemorrhage in an 18-year-old adult. Furthermore, the patient was now headache-free and did not have a decreased level of consciousness. Post-concussive syndrome was a possibility, but that became a diagnosis of exclusion given the other, more dangerous possible causes of the patient’s complaints.

**C**NS pathology: Non-traumatic etiologies in this category include stroke, hemorrhage, tumor and seizure. Stroke, hemorrhage and a space-occupying lesion are all unlikely, as you would expect to see focality in the neurologic examination. The patient’s family seemed attentive to her and no seizure activity was reported. Although focal seizures and a post-ictal state were possible, the duration of symptoms made this highly unlikely.

**H**ypoxia: This one is easy. The patient had a documented pulse oximetry (POx) of 99%. Although carbon monoxide poisoning can cause a normal POx reading in the setting of hypoxia, the patient had progressive symptoms in a variety of different settings. This is therefore removed as a possibility.

Vitamin **D**eficiencies: This category includes B12 and thiamine deficiencies. The patient was described as well developed and nourished, and there was no reported history of substance abuse. Although levels would need to be obtained to fully eliminate these as possibilities, these diagnoses are extremely unlikely. This category is therefore off of my list.

**E**ndocrinopathies: Here I had to consider disorders of the thyroid, parathyroid and adrenal glands, as well as diabetes mellitus. The thyroid stimulating hormone and calcium levels were within normal limits, eliminating thyroid and parathyroid disease. The sodium, potassium and glucose were also normal, eliminating both diabetes and an adrenal crisis.

**A**cute vascular disorders: Options in this category include shock, hypertensive encephalopathy and acute myocardial infarction. The patient’s complaints and hemodynamics do not support any of these etiologies. This category is off of my list.

**T**oxins, substance use, and medications: This category is broad and must be carefully considered. Although substances such as alcohol, anticholinergics and synthetic marijuana must be considered, the duration of her symptoms made all of these unlikely. For the patient’s symptoms to be ongoing and progressive over one week would require repeated exposures to these substances, including after she became confused. Her parents reported that she required redirection to complete tasks, so self-administration of substance is highly unlikely. This category is off my list.

**H**eavy metals: The patient did not work in industry or a laboratory. There is no reason, from the history, to think she had a heavy metal exposure.

I have eliminated all categories except for “infectious.” Now it was time for me to get out my magnifying glass and look at my clues a bit more closely. I started by reexamining the CSF results. The CSF had a WBC count of 12 cells/microL. With bacterial or viral meningitis, you anticipate counts of at least 100 cells/microL. Lower cell counts can, however, be seen with early bacterial meningitis, viral meningitis, neurosyphilis, tuberculous meningitis and encephalitis. The duration of symptoms, coupled with a negative gram stain, eliminated early bacterial meningitis. Given the normal glucose and protein on CSF analysis, tuberculous meningitis was excluded. Non-congenital neurosyphilis is extremely rare in this age group and a normal CSF protein essentially excludes this diagnosis. The normal protein also makes a viral etiology less likely. The patient’s profound mental status changes were much more suggestive of encephalitis, so I will continue to hunt for clues that support or refute this diagnosis.

We know the patient had just graduated from high school, so it is a reasonable assumption that her vaccinations were up to date. This minimizes the risk of her having encephalitis from measles, mumps, or rubella. It is important to remember that the patient also complained of abdominal pain and was recently diagnosed with a UTI. Could that “UTI” really have been a herpes simplex virus (HSV) outbreak? Considering this, HSV encephalitis is high on my list.

Of the non-infectious (aseptic) encephalitis etiologies, the one of greatest concern here is autoimmune. I am told there is a family history of autoimmune disease (lupus), which puts this patient at higher risk.

The two remaining possibilities are HSV encephalitis and autoimmune encephalitis. The abdominal pain, nausea and vomiting have yet to be explained and this, I believe, holds the key to the final diagnosis. Autoimmune disease can be triggered by viral infections, such as gastritis.

In review, this was an 18-year-old female with signs and symptoms consistent with encephalitis and a family history of autoimmune disease, who recently had a gastrointestinal illness. Bringing this all together, my final diagnosis is autoimmune encephalitis triggered by a systemic viral infection of the gastrointestinal tract. The diagnostic test is a magnetic resonance image (MRI) of the brain.

## CASE OUTCOME

**Image 1:**
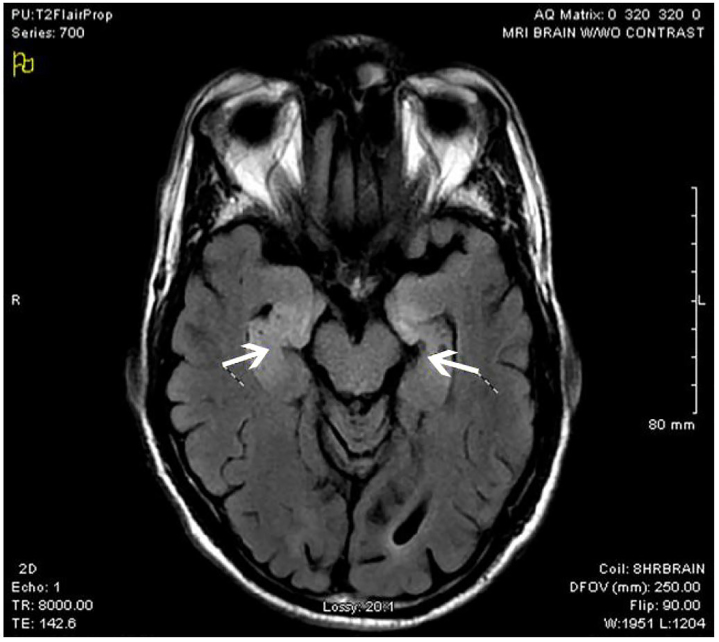
Axial T2 FLAIR sequence magnetic resonance imaging showing bilateral enhancement of the hippocampus (arrows). *FLAIR,* fluid attenuated inversion recovery

**Image 2:**
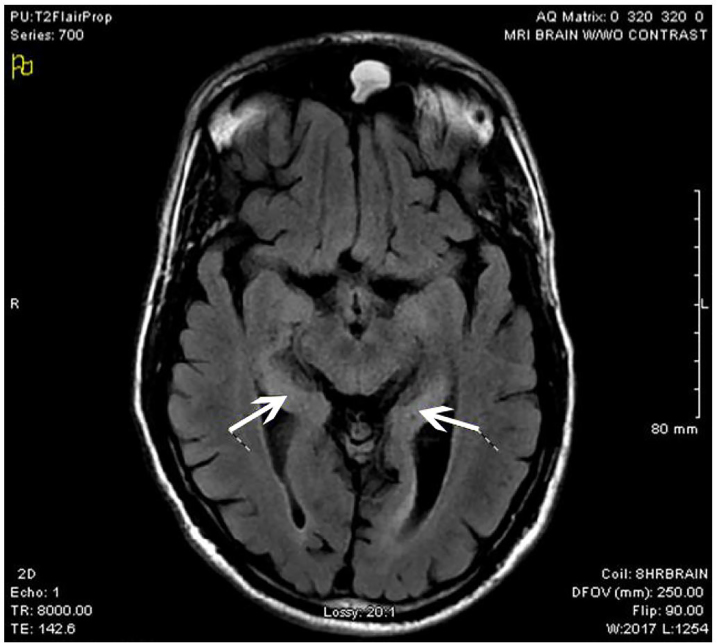
Axial T2 FLAIR sequence magnetic resonance imaging showing enhancement of the medial temporal lobe (arrows). *FLAIR,* fluid attenuated inversion recovery

The diagnostic study was an MRI of the brain. As described by the radiologist, the patient had patchy foci of T2 FLAIR hyperintensity and diffusion restriction, primarily involving the medial temporal lobes bilaterally, with additional smaller scattered areas throughout both cerebral hemispheres. Diagnostic considerations include encephalitis such as infectious or limbic/paraneoplastic (patient images shown in Images 1 and 2). Empiric vancomycin, ceftriaxone, and acyclovir were given and the patient was admitted to the intensive care unit (ICU). Her mental status continued to deteriorate, requiring mechanical ventilation as she began to have multiple seizures. The next day she began treatment for suspected autoimmune encephalitis with intravenous immunoglobulin (IVIG) methylprednisolone, and plasmapheresis. She began having refractory seizures on hospital day (HD) three, ultimately requiring levetiracetam, divalproex sodium, phenytoin, and pentobarbital drips. A tracheostomy tube was placed. On HD 18, a CSF autoantibody panel revealed autoantibodies to glutamic acid decarboxylase (GAD). The team planned to start rituximab therapy for GAD autoimmune encephalitis, but this was delayed by persistent fevers. On HD 29 she received her first dose of rituximab therapy, and on subsequent days she was able to be weaned from her antiepileptic regimen and gradually recovered neurologic function. On HD 34, the patient was weaned off of the ventilator. She was speaking through her Passy-Muir tracheostomy valve, texting, and laughing with family. She was transferred to rehab after her second dose of rituximab on HD 36. She was discharged home after her fourth and final dose of rituximab on HD 50. Per her family, she regained her cognitive baseline. Her tracheostomy was successfully reversed two weeks later.

## RESIDENT DISCUSSION

Encephalitis is an acute inflammation of the brain, and approximately 10% of the 20,000 cases seen in the United States annually are fatal.^1^–^3^ The primary injury occurs as the brain parenchyma suffers a widespread inflammatory response, causing edema, hemorrhage, and the destruction of neurons.

Data is limited in encephalitis as cases are rare and the testing required to make the diagnosis is expensive, slow, and inconsistently performed. Fifty percent of cases have no identifiable pathogen.^1^,^2^ When an etiology is identified, viruses are the most common cause.^4^ These viruses include HSV (10–14% of all cases worldwide), eastern and western equine encephalitis virus, West Nile virus, non-polio enteroviruses, varicella-zoster virus and rabies.^2^–^5^

Autoimmune-related etiologies are becoming recognized as an important cause of encephalitis and may account for up to one-third of cases.^6^ Autoimmune encephalitis can result from patients developing antibodies to voltage-gated potassium channels, N-methyl-D-aspartate (NMDA) receptors, gamma-aminobutyric acid (GABA) receptors, or glutamic acid decarboxylase (GAD) receptors.^5^,^7^

Autoimmune encephalitis tends to affect the limbic system (amygdala, hippocampus, hypothalamus), causing emotional lability, personality changes, and decreased memory.^8^ Patients may even have delusional or paranoid thoughts, visual or auditory hallucinations and seizures.^2^ It often progresses insidiously over a period of days to weeks, with patients eventually presenting to medical providers with seizures or headaches.^2^ Viral infections can set off a variety of inflammatory and autoimmune disorders such as systemic lupus erythematosus, Sjogren’s syndrome, Hashimoto thyroiditis and autoimmune encephalopathy.

The ED workup includes a set of basic labs, which often reveals a nonspecific elevation in the WBC count. Ammonia, carboxyhemoglobin level, toxicological screen, and blood cultures should all be measured to rule out alternative etiologies for the patient’s presentation. Studies of the CSF are often normal, regardless of the underlying etiology of the encephalitis. The most common abnormalities seen on CSF analysis are lymphocytic pleocytosis, an elevated protein level and oligoclonal bands.^8^,^9^ Computed tomography of the head is the first imaging study to order, as it will rule out other etiologies of altered mental status, such as space-occupying lesions and intracranial hemorrhage. MRI is the diagnostic imaging modality of choice as it is the most sensitive for encephalitis.^9^,^10^ T2-weighted and FLAIR sequences may show hyperintense signal and mild swelling of the medial temporal lobes,^8^ as it did in our patient.

Patients with encephalitis should therefore be admitted to the hospital for further evaluation, diagnostic studies, and management. Initial management in the ED includes supporting the patient’s airway, breathing, and circulation. Empiric antibiotics and acyclovir should be administered, since HSV encephalitis can have similar MRI findings.^8^ Seizures are common and are managed initially with benzodiazepines. Refractory seizures and status epilepticus can be treated with second- and third-line agents such as phenytoin, fosphenytoin, and phenobarbital. Inflammation and vasogenic edema caused by the encephalitis can lead to increased intracranial pressure that can be life threatening.^2^ Patients may require mannitol or hypertonic saline administration when there is a concern for elevated intracranial pressure, and the initial evaluation should include a measurement of the patient’s opening pressure on lumbar puncture. Patients should undergo additional CSF studies as part of their inpatient workup to differentiate between the various causes of encephalitis. Many CSF autoantibody studies may take days to weeks to result, and empiric treatment for autoimmune and infectious etiologies is appropriate preceding or in the absence of a confirmed diagnosis.

First-line treatment for autoimmune encephalitis consists of the administration of steroids (ex. methylprednisolone 30mg/kg/day) plus IVIG (0.4 g/kg/day) or plasmapheresis.^11^ Second-line treatment options include immunomodulators such as rituximab and cyclophosphamide.^11^,^12^,^13^ Patients with suspected autoimmune encephalitis should undergo a workup for underlying malignancy as a part of their admission.^14^ Both NMDA and GAD autoimmune encephalitis are associated with ovarian teratomas and gastrointestinal malignancies, respectively.

Prognosis for encephalitis is extremely variable based on the specific underlying etiology. Mortality for viral encephalitis ranges from <1% in cases of non-polio enteroviral encephalitis, to approximately 50% for eastern equine encephalitis, to nearly 100% for rabies encephalitis.^2^ Autoimmune encephalitis has a relatively good prognosis with proper treatment. There is a 10% mortality, and 81% of patients have favorable outcomes.^2^

## FINAL DIAGNOSES

Autoimmune limbic encephalitis due to glutamic acid decarboxylase antibodies, complicated by delirium and status epilepticus.

## TAKE-HOME POINTS

- Encephalitis is a life-threatening disease with many potential causes.
- History and physical examination are critical to making the diagnosis.
- Autoimmune encephalitis
  ○ Presents insidiously.
    ■ Personality changes, psychiatric symptoms, headaches, and seizures are the most common presenting complaints.
  ○ Lymphocytosis may be seen on CSF testing.
    ■ Neither sensitive nor specific.
  ○ CT imaging of the brain is a reasonable initial choice to rule out competing diagnoses.
  ○ MRI is the diagnostic imaging study of choice.
    ■ T2-weighted and FLAIR sequence images may show hyperintense signal and mild swelling of the medial temporal lobes.

## Figures and Tables

**Table 1:** Complete blood cell count of 18-year-old patient presenting with mental status changes, fever, and a family history of autoimmune disease.

Complete blood cell count	
White blood cells	6.6 K/mcL
Hemoglobin	12.4 g/dL
Hematocrit	36.8%
Platelets	251 K/mcL
Differential	
Polymorphonuclear leukocytes	52%
Lymphocytes	44%
Monocytes	3%
Eosinophils	1%

**Table 2:** Chemistry results.

Serum chemistries	
Sodium	141 mmol/L
Potassium	3.3 mmol/L
Chloride	104 mmol/L
Bicarbonate	27 mmol/L
Blood urea nitrogen	7 mg/dL
Magnesium	0.65mg/dL
Phosphorous	2.5 mg/dL
Total protein	7.1g/dL
Albumin	3.8 g/dL
Total bilirubin	0.7 mg/dL
Aspartate aminotransferase (AST)	26 u/L
Alanine aminotransferase (ALT)	25 u/L
Alkaline phosphatase	66 units/L
Additional Labs	
Thyroid stimulating hormone (TSH)	1.73 mIU/L
C-reactive protein (CRP)	0.34 mg/L
Erythrocyte sedimentation rate (ESR)	8 mm/hour
Coagulation Studies	
Prothrombin time (PT)	15.9 seconds
Partial thromboblastin time (INR)	31 seconds
International normalized ratio	1.2

**Table 3:** Urinalysis results.

Urinalysis	
pH	6.2
Specific gravity	1.020
Glucose	Negative
Ketones	Negative
Nitrites	2+
Leukocyte esterase	Negative
White blood cells	Trace
Red blood cells	0–5 count/uL

**Table 4:** Cerebrospinal fluid results.

Cerebrospinal fluid	
Glucose	75 mg/dL
Protein	29 mg/dL
White blood cells	12K count/uL
Red blood cells	3K count/uL
Polymorphonuclear leukocytes	17%
Lymphocytes	78%
Monocytes	5%
Gram stain	Negative
Opening pressure	12 cm H_2_O
